# Argyria from the Use of Nasal Drops
*A case report at a Clinical Pathological Conference held on February 19th, 1952, in the Department of Pathology, University of Bristol.


**Published:** 1952-10

**Authors:** 


### ARGYRIA FROM THE USE OF NASAL DROPS*

**Dr. C. D. Cross**—This woman, aged 58 at the time of her death, swallowed a fish-bone which stuck in her throat seventeen years ago. She thereafter developed a neurosis and periodically attended E.N.T. Out-Patients, where she was treated with argyrol nasal drops. This treatment was continued by her private doctor. During the past six years she attended Psychiatric Out-Patients also. Her complaints were numerous but nothing organic was discovered.

She was first seen in Bath in 1947, when she came to St. Martin's Hospital bearing a large series of envelopes containing numerous clinical reports and prescriptions. She was sent to the E.N.T. Clinic, because of a persistent cough, and a bronchoscopy was performed without result. She was then of normal colour. In January 1949 she was admitted for a further investigation by the surgeon but nothing was found, and her colour was normal. She took her own discharge after ten days. She was never examined by a physician and her blood pressure was not recorded. In November 1951 she was admitted unconscious, with a blood pressure of 250/140. I happened to see her in Casualty and her colour suggested argyria. She had oedema of legs and ankles. Blood urea was 140 mgm%. She recovered consciousness and lived until December 5th, when she died with signs of cerebral haemorrhage.

At autopsy the colour of the skin was a peculiar slaty-grey with a definite blue tinge. This affected the whole of the body but was especially noticeable in the face and neck. Death was due to a small cerebral haemorrhage, and there was left ventricle hypertrophy, some arteriosclerosis, and the finely scarred kidneys of essential hypertension. Pieces of tissue for histological examination were taken from the kidney and from the skin of the neck. They all showed a remarkable deposit of black grains along the collagen fibres particularly in the adventitia of blood vessels. There was a great deal in the dermis of the skin and in the basement membrane of the glomeruli and of the renal tubules. It had almost the appearance of a silver impregnation stain for reticulin.

It is interesting to note that she never had any silver medicament except the argyrol drops, which she had taken regularly for seven years.

**Mr. G. W. Griffin**—We sent some of the kidney to the Bristol City Analyst (Mr. E. G. Whittle) who reported that the black pigment was insoluble in acids and alkalis, dissolved in cyanide, and was proved spectrographically to be silver.

**Dr. J. A. Cosh**—Was the fish-bone ever found?

**Dr. C. D. Cross**—No. There was no evidence of any fish-bone. The trouble for which she was treated all that time was a neurosis.

**A questioner**—Are there many cases of argyria seen nowadays?

**Dr. F. G. Bolton**—Those people who live in the Westbury area may have seen an old woman walking about who has argyria.

**Professor T. F. Hewer**—I have never seen her but I have known a lady by sight for many years in another part of Bristol, and as it happens I was one day presented with part of her stomach by a local surgeon who performed a partial gastrectomy because of a duodenal ulcer. The serous coat of the stomach was an extraordinary grey colour. I recognized it as argyria—in fact, I might almost say I recognized the lady herself by the appearance of her stomach, because I asked the surgeon whether it came from this lady whom I knew by sight: I was delighted to discover I was correct! She had been treated with silver nitrate as a child, presumably because she had some sort of fits. It was fashionable in those times to treat epilepsy with silver nitrate. The histological appearance of her stomach was the same as that of the tissues in Dr. Cross's case.

* A case report at a Clinical Pathological Conference held on February 19th, 1952, in the Department of Pathology, University of Bristol.

**Dr. H. J. Bland**—There is a lady in Southmead now who has had argyria for seventeen years. She has a blood pressure of 240/160. She had selenium treatment some years ago. She sits in Southmead quite happily.

**Professor T. F. Hewer**—I suggest that a biopsy should be performed and a piece of skin taken to the City Analyst! Selenium does not produce pigmentation that could be confused with argyria.

**Dr. J. A. Cosh**—Does Professor Hewer haunt Cotham Hill? If so, he may have seen the pathetic figure of an old lady crippled with arthritis who has argyria and has also had selenium; she smells strongly of acetylene. What does your patient smell of, Dr. Bland?

**Dr. H. J. Bland**—Wintergreen!

**Professor A. V. Neale**—Why so malodorous? I should like to stress the danger of house physicians in out-patients giving " Rep. Mist." and saying " Off you go ".

**Dr. B. E. McConnell**—I have a fellow feeling and sympathy with the clinicians who had to deal with this lady. She was obviously poly-symptomatic and they must have wanted to get her out of their consulting rooms as soon as possible. It is most unfortunate that argyrol was given for so long a time, but it would be a pity if we dropped the use of argyrol altogether: like bromides, which are cheaper than phenobarbitone, it is good for a short period.

I have a patient with argyria of the conjunctivae due to argyrol drops. She is no longer receiving argyrol, at least not to my knowledge. It is confined to the conjunctivae.

**Professor T. F. Hewer**—I should like to add that argyria has nothing to do with hypertension, and the fact that in the present case, as in that of the old woman at Southmead, there happened to be hypertension is quite irrelevant. As far as I know argyria has only a cosmetic disadvantage. The occurrence of local argyria affecting the conjunctiva where the drops were applied in Dr. McConnell's case is interesting. Local argyria occurs in industry. We asked Dr. Cross to present this case because we have not had an autopsy on anyone with argyria, and we thought this example might remind people of the unwisdom of allowing silver preparations to be used over a long period.

